# Maternal Cadmium Exposure during Pregnancy and Size at Birth: A Prospective Cohort Study

**DOI:** 10.1289/ehp.1103711

**Published:** 2011-08-23

**Authors:** Maria Kippler, Fahmida Tofail, Renee Gardner, Anisur Rahman, Jena D. Hamadani, Matteo Bottai, Marie Vahter

**Affiliations:** 1 Institute of Environmental Medicine, Karolinska Institutet, Stockholm, Sweden; 2 International Centre for Diarrhoeal Disease Research, Bangladesh, Dhaka, Bangladesh

**Keywords:** arsenic, birth weight, cadmium, sex, urine

## Abstract

Background: Cadmium (Cd) is an embryotoxic and teratogenic metal in a variety of animal species, but data from humans are limited.

Objectives: The aim of the present study was to assess the effects of maternal Cd exposure in pregnancy on size at birth.

Methods: This prospective cohort study was nested in a population-based nutritional supplementation trial in pregnancy conducted in rural Bangladesh. We selected women recruited from February 2002 through January 2003 who had a singleton birth with measurements of size at birth and had donated a urine sample in early pregnancy for Cd analyses (*n* = 1,616). Urinary Cd was measured with inductively coupled plasma mass spectrometry and adjusted for specific gravity.

Results: Multiple linear regression analyses adjusted for sex and other potential confounders showed that maternal urinary Cd (median, 0.63 μg/L) was significantly negatively associated with birth weight [unstandardized regression coefficient B = –31.0; 95% confidence interval (CI): –59, –2.8] and head circumference (B = –0.15; 95% CI: –0.27, –0.026). However, associations appeared to be limited to girls, with little evidence of effects in boys. A 1-μg/L increase in Cd in maternal urine was associated with a 0.26-cm (95% CI: –0.43, –0.088 cm) and 0.24-cm (95% CI: –0.44, –0.030 cm) decrease in girls’ head and chest circumferences, respectively, and a 45-g (95% CI: –82.5, 7.3 g) decrease in birth weight. Quantile regression analyses indicated that associations with maternal Cd were similar for girls of smaller (25th percentile) and larger (50th and 75th percentiles) sizes at birth.

Conclusion: We found evidence of a sex difference in the association between maternal Cd exposure and birth size, which was apparent only in girls. Results add support for the need to reduce Cd pollution to improve public health.

Human exposure to the toxic metal cadmium (Cd) occurs mainly through food, such as cereals, seafood, and offal, and inhalation of tobacco smoke (Järup and Åkesson 2009). Once absorbed, Cd has a long half-life in the body, especially in the kidneys. Chronic Cd exposure has been shown to adversely affect kidney and bone and to increase the risk of cancer (Straif et al. 2009) and overall mortality (Järup and Åkesson 2009). Cd also functions as an endocrine disruptor (Ali et al. 2010; Johnson et al. 2003) and may thus affect reproduction and child development (Henson and Chedrese 2004). In general, women are more susceptible to Cd toxicity, mainly because of increased intestinal uptake of Cd given low iron stores (Åkesson et al. 2002; Berglund et al. 1994; Nishijo et al. 2004a; Vahter et al. 2007), which are more prevalent in women than in men.

There is, however, little information on the effects of Cd exposure in early life. Cd has been shown to be both embryotoxic and teratogenic in a variety of animal species (Thompson and Bannigan 2008), but this has not yet been confirmed in humans. Cd accumulates in human placenta (Kippler et al. 2010a; Osman et al. 2000), but the placenta is not a complete barrier, and Cd concentrations in cord blood increase with maternal exposure (Kippler et al. 2010a). There is increasing evidence of associations between maternal Cd exposure and adverse pregnancy outcomes, such as reduced size at birth (Galicia-Garcia et al. 1997; Llanos and Ronco 2009; Nishijo et al. 2004b; Salpietro et al. 2002; Shirai et al. 2010; Zhang et al. 2004) and preterm delivery (Nishijo et al. 2002). However, studies that have reported associations included very few women (44–78 women), and associations with the same outcomes varied markedly among the studies. Residual confounding cannot be excluded because very few covariates were considered. In contrast, two larger studies (106–262 women) found no association between maternal Cd exposure and size at birth (Odland et al. 1999; Osman et al. 2000).

In the present study, we took advantage of our large, population-based, longitudinal mother–child cohort in Bangladesh to assess the effects of maternal Cd exposure on size at birth. We previously reported that arsenic (As) exposure is associated with reduced size at birth (Rahman et al. 2009). Additionally, pregnant women in this rural area had elevated concentrations of Cd in their placentas, and placental Cd was inversely associated with zinc (Zn) in cord blood, suggesting a possible effect of Cd on the transfer of Zn to the fetus (Kippler et al. 2010a). Thus, it is possible that Cd has adverse effects on pregnancy outcomes as well.

## Materials and Methods

*Study area and design.* The present study was nested in a population-based food and micronutrient supplementation trial in pregnancy in women in rural Bangladesh [Maternal and Infant Nutrition Interventions, Matlab (MINIMat)], which enrolled approximately 4,500 pregnant women from November 2001 through October 2003, as described previously (Tofail et al. 2008). During monthly household visits, community health research workers offered a pregnancy urine test to women who had missed their menstrual period. In the case of a positive pregnancy test, the women were asked to donate a urine sample for future analysis and thereafter were invited to the nearest health center for further examination. The present study included the women who were identified as pregnant during one calendar year, from 1 February 2002 through 31 January 2003, who were enrolled in the MINIMat trial and had a singleton birth with measurements of size at birth. In total, 1,697 women fulfilled these criteria (Rahman et al. 2009).

The study has been approved by the research and ethical review committee at the International Centre for Diarrhoeal Disease Research, Bangladesh (ICDDR,B), as well as the regional ethics committee at Karolinska Institutet, Sweden. Oral and written consents were obtained from the women.

*Exposure assessment.* Of the 1,697 women, 1,616 donated a urine sample, on average at gestational week 8, and this urine sample was used for assessment of Cd exposure. Urinary Cd is a well-documented biomarker of chronic exposure (Järup and Åkesson 2009). These 1,616 women did not significantly differ in age, body mass index (BMI), socioeconomic status (SES), or parity, or with regard to characteristics of their newborns (birth weight, length, head and chest circumference) from those who did not provide a urine sample (*n* = 81). However, those who donated a sample had slightly longer gestation (median, 39.4 weeks) than those who did not (median, 39.0 weeks; *p* = 0.011).

Cd in urine was measured with inductively coupled plasma mass spectrometry (ICPMS; model 7500ce; Agilent Technologies, Waldbronn, Germany) at Karolinska Institutet (Stockholm, Sweden). Samples were stored at –70°C in acid-washed plastic vials. The ICPMS was operated in helium mode and ^111^Cd was monitored. The sample preparation and analytical methods have been described elsewhere (Kippler et al. 2007, 2009). No samples were below the limit of detection (LOD; < 0.02 μg/L). Quality control was assessed by analyses of two commercial control materials (Seronorm^TM^ Trace Elements Urine Blank, ref. 201305, lot OK4636, and Seronorm^TM^ Trace Elements Urine, ref. 201205, lot NO2525; SERO AS, Billingstad, Norway). The obtained mean (± SD) urinary Cd concentrations in the two control materials were 0.26 ± 0.05 μg/L (recommended value 0.31 ± 0.05) and 4.8 ± 0.14 μg/L (5.06 ± 0.22), respectively. We have previously measured the sum of inorganic As and its methylated metabolites, referred to as urinary As, in these women because many are highly exposed to As via drinking water (Vahter et al. 2006). These measurements were performed with hydride generation atomic absorption spectroscopy (Vahter et al. 2006). To compensate for variation in urine dilution, the final Cd and As concentrations were adjusted to the mean specific gravity (1.012 g/mL) of the urine samples (Nermell et al. 2008).

*Outcomes and covariates.* Birth weight was measured with electronic or beam scales with precision of 10 g (UNICEF Uniscale; SECA Gmbh & Co., Hamburg, Germany), length with a locally produced wooden length board (accurate to 1 mm), and head and chest circumference with a flexible nonstretchable measuring tape (accurate to 1 mm) (Rahman et al. 2009). Approximately 40% of the women delivered at the health clinics and had birth anthropometry measured in connection with the delivery. A birth notification system was established for women who delivered at home, and birth anthropometry was most often measured within 24 hr. If more than 72 hr had passed since delivery, the weight was adjusted as described in detail elsewhere (Arifeen et al. 2000). Gestational age was calculated by subtracting the date for the last menstrual period from the date of birth. The date of last menstrual period was obtained by interviewing the women immediately after identification of pregnancy. For women who had forgotten this date (*n* = 26), we used the last menstrual period estimated by ultrasound measurements (Rahman et al. 2009).

Covariate information was obtained during household visits by the health research workers and at routine visits at the health facilities, as well as from the Health and Demographic Surveillance System database maintained by ICDDR,B (Dhaka, Bangladesh). Maternal height and weight were recorded at the health facilities, approximately in gestational week 8. Education was defined as years of formal schooling and categorized as no education, < 5 years, ≥ 5 to < 10 years, and ≥ 10 years. SES was classified according to a wealth index based mainly on household assets, as described previously (Gwatkin et al. 2000). Maternal tobacco smoking and betel/tobacco chewing during pregnancy were defined as never or ever. The season of birth was categorized as premonsoon (January–May), monsoon (June–September), or postmonsoon (October–December).

*Statistical analyses.* The statistical analyses were performed with Statistica for Windows (version 9.1; StatSoft, Inc., Tulsa, OK, USA) and STATA (version 11; StataCorp., College Station, TX, USA). All tests were two sided, and a *p* < 0.05 was considered statistically significant. Bivariate analyses between exposures, sizes at birth, and covariates were evaluated by Spearman’s correlation coefficient or analysis of variance depending on the type of data analyzed. The *p*-value for the difference between two independent groups was determined with the Mann–Whitney *U*-test.

We further assessed associations between maternal urinary Cd (nontransformed) and size at birth (birth weight, birth length, head and chest circumference) using multivariable-adjusted linear regression analyses. The residual analyses indicated no major deviation from the assumptions of linear regression. We adjusted for variables significantly associated with either size at birth or urinary Cd (*p* < 0.05) in the bivariate analyses or that had previously been associated with size at birth. Maternal age and parity, and maternal education and SES were highly correlated (*r*_s_ > 0.50). Therefore, we adjusted for maternal age and SES only, because they were more strongly associated with outcomes than were parity and maternal education. We evaluated colinearity by tolerance and variance inflation factor in the linear regression analyses. The final multiple linear regression analyses were adjusted for maternal age, BMI, SES, hemoglobin in gestational week 14, urinary As at gestational week 8, betel use, season of birth, gestational age at birth, and sex. Additionally, we stratified by sex and by SES (median split) and repeated the above analysis. Also, we assessed whether adding the different food (early and usual) and/or micronutrient supplementations (three different types) into the models modified the association between Cd and size at birth. We used “seemingly unrelated estimation” in STATA to adjust for conducting multiple tests on a set of correlated outcomes dealing with size at birth and compared these model estimates with estimates from the analysis described above.

To assess the effect of maternal urinary Cd on the whole distribution of sizes at birth, we performed multivariable-adjusted quantile regression analyses using STATA. Linear regression models the relationship between a set of predictor variables and the mean of the response variable. Quantile regression models the relationship between a set of predictor variables and specific percentiles (or quantiles) of the response variable. Thus, quantile regression allows us to estimate associations between an exposure and the outcome at different levels of the outcome distribution, instead of associations with the mean value of the outcome only, and thus provides a method to determine whether a predictor variable, such as Cd, may have a different association with birth size among children with low birth size compared with children with normal birth size (Bottai et al. 2010). Additionally, quantile regression estimates are robust to outliers in the data set. We assessed the 25th percentile (lowest quantile), 50th percentile (median quantile), and 75th percentile (highest quantile) for the different birth outcomes. Regression models for all three quantiles were estimated jointly using simultaneous quantile regression in STATA. Estimates, confidence intervals (CIs), and *p*-values were based on 200 bootstrap samples. The Wald test was used to test for difference between the jointly estimated regression coefficients across different quantiles.

## Results

The background characteristics and biomarkers of the mothers are presented in Table 1. Of the 1,616 mothers, 75% had urinary Cd concentration < 1 μg/L. Maternal age ranged from 14 to 45 years, with an average of 26 years. About one-third of the women had a BMI < 18.5 kg/m^2^. None of the women reported smoking during pregnancy, but approximately 38% reported betel chewing. About one-third of the women were primiparous, and the multiparous women had on average two children (range, one to eight). Table 1 also shows the descriptive data of the newborns; 52% of the newborns were boys, the average gestational age was 39 weeks (range, 29–45 weeks), and around 8% were born preterm (< 37 weeks). Birth weight ranged from 1,059 g to 4,000 g, with an average of 2,672 g. After excluding those born preterm, 38% still had a low birth weight (< 2,500 g).

**Table 1:** Characteristics of the mothers and their newborns.

Characteristic	*n*	Mean ± SD (range) or percent	Median	25th–75th percentile
Maternal								
Age (years)		1,616		27 ± 6.0 (14–45)		26		22–31
Weight (kg)*a*		1,612		45 ± 6.6 (25–74)		44		40–49
Height (cm)		1,616		150 ± 5.2 (134–170)		150		146–153
BMI (kg/m^2^)*a*		1,612		20 ± 2.6 (13–31)		20		18–21
< 18.5		488		30				
≥ 18.5		1,127		70				
Parity		1,616		1.4 ± 1.4 (0–8)		1		0–2
Primiparous		529		33				
Multiparous		1,087		67				
Urinary Cd (μg/L)*a*		1,616		0.81 ± 0.67 (0.044–7.0)		0.63		0.38–1.0
Urinary As (μg/L)*a*		1,509		152 ± 175 (1.2–1,235)		80		37–206
Hemoglobin (g/dL)*b*		1,504		11.6 ± 1.3 (8.0–17.3)		11.6		10.8–12.4
Tobacco smoking (yes/no)		1,562		(0/100)				
Betel chewing (yes/no)		1,562		38/62				
Newborns								
Sex (male/female)		1,616		52/48				
Gestational age (weeks)		1,611		39 ± 1.8 (29–45)		39		38–40
< 37		130		8				
≥ 37		1,481		92				
Weight (g)		1,616		2,672 ± 414 (1,059–4,000)		2,677		2,419–2,930
< 2,500		520		32				
≥ 2,500		1,096		68				
Length (cm)		1,603		48 ± 2.3 (38–60)		48		47–49
Head circumference (cm)		1,608		32 ± 1.6 (24–40)		33		31–34
Chest circumference (cm)		1,608		31 ± 2.2 (22–53)		31		30–33
**a**Gestational week 8. **b**Gestational week 14.								

In the bivariate analyses, maternal urinary Cd was not significantly correlated with birth weight, length, and head or chest circumference but was positively correlated with maternal age and urinary As concentrations and negatively correlated with maternal education, SES, and hemoglobin (Table 2). In addition, birth outcomes were significantly correlated with gestational age at birth and with maternal height, weight, BMI, age, parity, SES, and level of education (Table 2).

**Table 2:** Spearman’s rank correlation coefficients (*p*-value) of associations between exposures, maternal covariates, and child anthropometry measures at birth.

Child measures							Maternal urinary Cd (μg/L)
Variable	Weight (g)	Length (cm)	Head circumference (cm)	Chest circumference (cm)			
Urinary Cd		–0.011 (0.65)		0.014 (0.57)		–0.029 (0.24)		–0.040 (0.11)		—
Urinary As		–0.0067 (0.80)		–0.036 (0.16)		–0.066 (0.011)		–0.067 (0.0090)		0.088 (0.0059)
Hemoglobin		–0.0059 (0.82)		–0.000079 (0.99)		0.029 (0.26)		0.033 (0.20)		–0.059 (0.021)
Age		0.099 (< 0.001)		0.083 (< 0.001)		0.050 (0.045)		0.067 (0.0075)		0.22 (< 0.001)
Parity*a*		0.13 (< 0.001)		0.069 (0.043)		0.044 (0.10)		0.052 (0.039)		0.18 (< 0.001)
Height		0.24 (< 0.001)		0.27 (< 0.001)		0.16 (< 0.001)		0.19 (< 0.001)		–0.046 (0.062)
Weight		0.32 (< 0.001)		0.27 (< 0.001)		0.24 (< 0.001)		0.28 (< 0.001)		–0.024 (0.34)
BMI		0.24 (< 0.001)		0.17 (< 0.001)		0.18 (< 0.001)		0.22 (< 0.001)		–0.0098 (0.69)
SES		0.13 (< 0.001)		0.17 (< 0.001)		0.13 (< 0.001)		0.15 (< 0.001)		–0.19 (< 0.001)
Education*b*		0.10 (< 0.001)		0.15 (< 0.001)		0.068 (< 0.001)		0.10 (< 0.001)		–0.17 (< 0.001)
Gestational age		0.40 (< 0.001)		0.43 (< 0.001)		0.37 (< 0.001)		0.36 (< 0.001)		–0.0062 (0.80)
**a**Parity (primiparous vs. multiparous). **b**Education (no education, < 5 years education, ≥ 5 to < 10 years, ≥ 10 years).										

Urinary Cd did not differ between mothers of infants born preterm (median, 0.61 μg/L) and those born at term (0.62 μg/L; *p* = 0.77). Multiparous women had significantly higher urinary Cd concentrations (median, 0.70 μg/L) than did primiparous women (median, 0.50 μg/L; *p* < 0.001), but they were also older than the primiparous women (median, 29 vs. 21 years of age; *p* < 0.001). Betel chewing was not significantly associated with maternal urinary Cd or any of the birth outcomes (data not shown).

In girls, urinary Cd was negatively associated with chest circumference (*r*_s_ = –0.071, *p* = 0.048), birth weight (*r*_s_ = –0.055, *p* = 0.13), and birth length (*r*_s_ = –0.041, *p* = 0.25). In boys, urinary Cd was positively associated with birth length (*r*_s_ = 0.068, *p* = 0.050) and birth weight (*r*_s_ = 0.027, *p* = 0.43) and weakly associated with reduced chest circumference (*r*_s_ = –0.014, *p* = 0.68). Head circumference was not correlated with maternal urinary Cd in girls or boys (*r*_s_ = –0.0072, *p* = 0.25; and *r*_s_ = –0.0072, *p* = 0.83, respectively.)

Multivariable-adjusted linear regression analyses, including maternal age, BMI, SES, hemoglobin, urinary As, and betel use and infant season of birth, gestational age, and sex, showed significant associations of maternal urinary Cd with mean birth weight (unstandardized regression coefficient B = –31.0; 95% CI: –59, –2.8; *p* = 0.031; Table 3) and mean head circumference (B = –0.15; 95% CI: –0.27, –0.026; *p* = 0.018). Moreover, maternal urinary Cd was close to negatively associated with mean chest circumference (B = –0.14; 95% CI: –0.30, 0.021; *p* = 0.088), whereas no association was found with birth length. Sex was significant in all models, confirming that, on average, girls were smaller than boys (*p* < 0.001 for all outcomes). When a multiplicative interaction term between urinary Cd and sex was included in the multivariable-adjusted regression analyses (Table 3), the interaction term was not significantly associated with any of the birth outcomes (*p* > 0.05). The stratified analyses showed that the inverse associations between maternal urinary Cd and mean birth outcomes were evident only in girls (Table 3), whereas in boys, maternal urinary Cd was not associated with any of the birth outcomes. Evaluation of effect size with linear regression analysis showed that a 1-μg/L increase in maternal urinary Cd was associated with a decrement of 45 g (95% CI: –82.5, –7.3) in mean birth weight, 0.26 cm (95% CI: –0.43, –0.088) in mean head circumference, and 0.24 cm (95% CI: –0.44, –0.030) in mean chest circumference in girls. Results were consistent with those shown when associations were estimated using seemingly unrelated postestimation to account for multiple testing on correlated outcomes (*p*-values shown in Table 3). We further stratified for high and low SES (median split) and found that the effect estimates of Cd did not change significantly in girls or boys (data not shown).

**Table 3:** Multivariable*^a^* linear regression analyses of the association between maternal urinary Cd in gestational week 8 and size at birth.

All			Boys (*n* = 712)			Girls (*n* = 670)		
Predicted	B (95% CI)	*p*-Value	Adjusted *p*-value*b*	B (95% CI)	*p*-Value	Adjusted *p*-value*b*	B (95% CI)	*p*-Value	Adjusted *p*-value*b*	Interaction *p*-value*c*
Birth weight (g)		–31.0 (–59, –2.8)	0.031	0.029		–17.0 (–59.4, 25.4)	0.43	0.46		–44.9 (–82.5, –7.3)	0.020	0.011		0.19
Birth length (cm)		–0.043 (–0.21, 0.12)	0.61	0.58		0.071 (–0.18, 0.32)	0.58	0.54		–0.14 (–0.36, 0.077)	0.20	0.14		0.11
Head circumference (cm)		–0.15 (–0.27, –0.026)	0.018	0.017		–0.051 (–0.23, 0.13)	0.58	0.58		–0.26 (–0.43, –0.088)	0.0031	0.0019		0.12
Chest circumference (cm)		–0.14 (–0.30, 0.021)	0.088	0.083		–0.071 (–0.32, 0.18)	0.58	0.59		–0.24 (–0.44, –0.030)	0.025	0.019		0.25
**a**Adjusted for maternal age, BMI, SES, hemoglobin at gestational week 14, urinary As at gestational week 8, and betel use (never/ever) and infant season of birth (premonsoon, monsoon, and postmonsoon) and gestational age (< 37 weeks = 1; ≥ 37 weeks = 0). Estimates for boys and girls combined were also adjusted for sex. **b***p*-Value after seemingly unrelated estimation, to adjust for conducting multiple tests on correlated outcomes. **c***p*-Value for the interaction term between maternal urinary Cd and sex (described in text).														

We also performed multivariable-adjusted linear regression analyses with gestational age at birth as the dependent variable, adjusting for all potential confounders. There was no significant association with maternal urinary Cd in the cohort as a whole, or in girls or boys (data not shown). Adding the different food and micronutrient supplementations that were ingested during pregnancy into the models did not modify the associations between Cd and size at birth (data not shown), even when the analysis was restricted to the girls.

**Figure 1:**
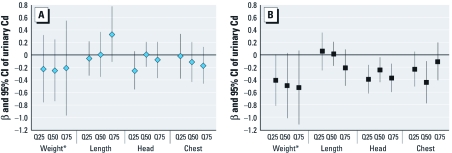
β-Coefficients and 95% CIs for maternal urinary Cd in each quantile [25th (Q25), 50th (Q50), and 75th (Q75)] of birth weight, length, and head and chest circumference for boys (*A*) and girls (*B*). In boys, there were significant differences between the 25th and 50th quantile specific estimates (*p* = 0.046) for head circumference, and in girls, there was a significant difference between the 50th and 75th quantile specific estimates (*p* = 0.030) for chest circumference. No other significant differences between any of the specific quantile estimates were detected (*p* > 0.05). *Birth weight has been divided by 100 to fit the scale of the figure.

The multivariable-adjusted quantile regression analysis confirmed that maternal urinary Cd was associated with birth size in girls but not in boys (Figure 1). Moreover, the results indicate that the associations between maternal urinary Cd and size at birth were very similar across the different percentiles of each outcome.

## Discussion

The present study, which to our knowledge is the largest to estimate effects of Cd on fetal development, shows an unexpected difference in the association between maternal Cd exposure during pregnancy and infant size at birth by sex, with inverse associations in girls but little evidence of an effect in boys. The strongest associations were found for head circumference and birth weight. A 1-μg/L increase in Cd in maternal urine during pregnancy was associated with a decrease in head circumference of 0.26 cm and in birth weight of 45 g in girls. The magnitude of the estimated effects is not necessarily of clinical importance for individual children but may have considerable public health relevance given the high prevalence of elevated exposure to Cd, mainly through the diet.

The main strengths of this study include the prospective study design and the large sample size with a wide range of Cd exposure. Also, we had extensive data on potential confounders, including maternal age, nutrition, season of birth, and SES. We also adjusted for As exposure and betel chewing, which have previously been associated with size at birth (Rahman et al. 2009; Senn et al. 2009). However, we cannot rule out the possibility that our findings may still be biased by unmeasured confounders or by residual confounding. We assessed individual maternal Cd exposure by concentrations in urine, a well-recognized biomarker of chronic Cd exposure (Järup and Åkesson 2009). Urinary Cd concentrations were adjusted for specific gravity, because creatinine-adjustment is highly dependent on body composition and type of diet (Nermell et al. 2008), which may also be associated with birth weight. The analytical measurements were performed with an ICPMS method with high sensitivity and efficient removal of potential interferences (e.g., molybdenum). We selected all the women who were identified as pregnant during one calendar year, to cover potential temporal variation, especially in size at birth. Furthermore, none of the women smoked, which otherwise would have been a major confounder, because smoking is associated with several adverse outcomes of pregnancy, including low birth weight (DiFranza and Lew 1995), as well as increased Cd exposure (Olsson et al. 2002).

We tested the robustness of our findings using both conventional multivariate linear regression models and quantile regression models. The quantile regression estimates for the median generally agreed with the linear regression estimates in terms of direction and magnitude, indicating that the linear regression estimates were not unduly influenced by outlying values. We did not find any evidence that Cd differentially affected children of smaller size.

A limitation of the study is that we did not measure Cd in cord blood, which might have provided more precise information of the prenatal Cd exposure. Two previous studies, although limited in size (49 and 45 women), found inverse associations between cord blood Cd concentrations and birth weight (Galicia-Garcia et al. 1997; Salpietro et al. 2002). Another study reported a significant inverse association with head circumference, but not with birth weight (Lin et al. 2010). However, the effect on fetal growth may at least partly be related to the accumulation of Cd in placenta because it is associated with impaired transport of nutrients to the fetus. Indeed, placental Cd concentrations have been negatively associated with size at birth in a few small studies (Llanos and Ronco 2009; Ronco et al. 2005). Maternal urinary Cd is associated with both placenta and cord blood Cd (Kippler et al. 2010a) and would be a useful exposure measure for evaluating combined effects of direct fetal Cd exposure and indirect effects due to placental accumulation of Cd. Maternal urinary Cd concentrations were measured on only one occasion in early pregnancy, but urinary Cd concentrations have previously been shown to be rather constant throughout pregnancy (Hernandez et al. 1996).

There are several possible mechanisms by which maternal Cd exposure directly or indirectly may affect size at birth. We have previously shown that pregnant women in Matlab had elevated Cd concentrations in their placentas and that these Cd concentrations were inversely association with Zn in cord blood, suggesting that Cd may disturb Zn transfer to the fetus (Kippler et al. 2010a). Before that, an interaction between Cd and Zn in the placenta, leading to decreased Zn transfer to the fetus, had been shown only *in vitro* and in women with increased Cd exposure via smoking (Kuhnert et al. 1987; Wier et al. 1990). Because insufficient Zn transfer to the fetus is likely to have consequences for pregnancy outcomes, especially in terms of intrauterine growth retardation and preterm delivery (Jameson 1993), this has been suggested as one of the main mechanisms for the Cd-related reduction in birth weight (Kippler et al. 2010a; Kuhnert et al. 1987; Ronco et al. 2005). However, because we found a sex difference in the association between maternal urinary Cd and size at birth, that mechanism may only partly explain the present findings. Another possible explanation for our findings may be the recently identified endocrine-disrupting properties of Cd.

Glucocorticoid exposure *in utero* has been associated with impaired fetal growth (McTernan et al. 2001). A key regulator in the fetoplacental barrier toward maternal glucocorticoids is 11β-hydroxysteroid dehydrogenase type 2 (11β-HSD2), which inactivates maternal cortisol to cortisone (Seckl and Holmes 2007). In fact, it has been shown that placental 11β-HSD2 gene expression is reduced in pregnancies with intrauterine growth restriction, and small-for-gestational-age babies appear to have elevated cortisol concentrations (McTernan et al. 2001). However, the underlying mechanism is unknown. Cd seems to be one factor affecting 11β-HSD2; 1 μM CdCl_2_ (cadmium chloride) markedly reduced *11*β-*HSD2* gene activity, mRNA, and protein levels in cultured human trophoblastic cells *in vitro* (Yang et al. 2006). On the other hand, very high Cd exposure of pregnant rats (50 mg/L in drinking water) did not alter placental 11β-HSD2, but corticosterone concentrations (corresponding to cortisol in humans) increased and the pup weights decreased (Ronco et al. 2009). Notably, the effect of prenatal glucocorticoid exposure appears to be sex specific. In mothers receiving glucocorticoid treatment for asthma, 11β-HSD2 activity was significantly decreased in placentas of female but not male fetuses, and this was associated with increased umbilical cord blood cortisol levels and reduced birth weight (Murphy et al. 2003). Thus, decreased 11β-HSD2 activity and increased concentrations of cortisol may be fundamental features in female fetal growth impairment (Clifton and Murphy 2004).

Cd may also interfere with the insulin-like growth factor (IGF) axis and thereby reduce fetal growth in a sex-specific manner. Rats given drinking water with 50 mg Cd/L had lower concentrations of IGF-1 and insulin-like growth factor-binding protein 3 (IGFBP-3) than did controls (Turgut et al. 2005). In human pregnancies, both IGF-1 and IGFBP-3 are usually positively associated with birth weight (Murphy et al. 2006). Furthermore, two epidemiologic studies have found that IGF-1 and IGFBP-3 levels in umbilical cord blood/plasma were higher in female than in male infants (Geary et al. 2003; Vatten et al. 2002).

We also hypothesize that Cd may affect the sex-specific programming, which begins very early in pregnancy and may contribute to the vulnerability of the developing fetus to maternal stressors (Mueller and Bale 2008). In an experimental study on chick embryos, Cd was found to down-regulate gene expression (levels) of DNA methyltransferases regulating the *de novo* DNA methylation, which is essential for normal embryogenesis (Doi et al. 2011). Interestingly, female embryonic stem cells were found to lose DNA methylation at a much faster rate than did male stem cells (Ooi et al. 2010).

Low birth weight is a significant public health problem worldwide, and it has been associated with neonatal mortality and morbidity, as well as increased risk for the development of diseases later in life (Murphy et al. 2006). The association between Cd and reduced birth weight in girls (an estimated decrease of 45 g for a 1-μg/L increase in maternal urinary Cd) corresponds to about one-quarter of the estimated effect of smoking during pregnancy on birth weight (~ 200 g lower birth weight for mothers who smoke than for nonsmoking mothers) (Rogers 2009), part of which may be mediated via the Cd inhaled with the tobacco smoke. To further put the effect size into perspective, an increase in birth weight of 37 g was achieved by supplementation with folic acid and iron in a double-blind randomized community trial in rural Nepal (Christian et al. 2003). In the present study, associations between Cd and birth size outcomes in girls were independent of SES and similar in small and larger infants; however, the relevance of our findings for birth outcomes in better-nourished populations needs to be evaluated.

We believe that the main source of Cd in this population is the diet, especially plant-derived foods, because none of the women smoked. The median maternal urinary Cd concentration (median, 0.63 μg/L) was higher than that reported in young women in the United States (geometric mean, 0.27 μg/g creatinine) and Europe (median, 0.31 μg/L) (Åkesson et al. 2002; McElroy et al. 2007), probably due to the rice-based diet, because rice is known to take up Cd from the soil to a greater extent than many other plants. Rice samples collected from 63 families in the study area contained on average 69 μg Cd/kg dry weight (10th and 90th percentiles, 11–98 μg Cd/kg) (Kippler et al. 2010b), which would correspond to a daily intake of 25–35 μg Cd, assuming a daily consumption of 400–500 g rice grains for an adult person (Khan et al. 2009). This daily Cd intake is similar to that in other Asian countries where rice is the main staple food and also to that in most individuals consuming vegetarian diets (European Food Safety Authority 2009; Ikeda et al. 2010). It is essential to investigate other potential sources of Cd, particularly vegetables and wheat, as well as agricultural fertilizers that may be a major contributor to Cd in agricultural soil.

## Conclusions

We found significant inverse associations between maternal Cd exposure and birth anthropometry in girls, especially head circumference and birth weight, but no associations in boys. These findings, along with evidence from previous experimental and observational studies, suggest that there may be sex differences in both toxicokinetics (i.e., intestinal uptake) and toxicodynamics of Cd. It is essential to follow the children through childhood to clarify whether the apparent effect on growth remains and whether the early-life Cd exposure is associated with other health outcomes later in childhood. This study clearly emphasizes the need to consider early-life effects of Cd in health risk assessments and to reduce Cd pollution worldwide.

## References

[r1] Åkesson A, Berglund M, Schutz A, Bjellerup P, Bremme K, Vahter M. (2002). Cadmium exposure in pregnancy and lactation in relation to iron status.. Am J Public Health.

[r2] Ali I, Penttinen-Damdimopoulou PE, Makela SI, Berglund M, Stenius U, Åkesson A (2010). Estrogen-like effects of cadmium *in vivo* do not appear to be mediated via the classical estrogen receptor transcriptional pathway.. Environ Health Perspect.

[r3] Arifeen SE, Black RE, Caulfield LE, Antelman G, Baqui AH, Nahar Q (2000). Infant growth patterns in the slums of Dhaka in relation to birth weight, intrauterine growth retardation, and prematurity.. Am J Clin Nutr.

[r4] Berglund M, Åkesson A, Nermell B, Vahter M. (1994). Intestinal absorption of dietary cadmium in women depends on body iron stores and fiber intake.. Environ Health Perspect.

[r5] Bottai M, Cai B, McKeown RE (2010). Logistic quantile regression for bounded outcomes.. Stat Med.

[r6] Christian P Khatry SK Katz J Pradhan EK LeClerq SC Shrestha SR 2003 Effects of alternative maternal micronutrient supplements on low birth weight in rural Nepal: double blind randomised community trial. BMJ 326 571; doi: [Online 15 March 2003] 10.1136/bmj.326.7389.571 12637400 PMC151518

[r7] Clifton VL, Murphy VE (2004). Maternal asthma as a model for examining fetal sex-specific effects on maternal physiology and placental mechanisms that regulate human fetal growth.. Placenta.

[r8] DiFranza JR, Lew RA (1995). Effect of maternal cigarette smoking on pregnancy complications and sudden infant death syndrome.. J Fam Pract.

[r9] Doi T, Puri P, McCann A, Bannigan J, Thompson J. (2011). Epigenetic effect of cadmium on global de novo DNA hypomethylation in the cadmium-induced ventral body wall defect (VBWD) in the chick model.. Toxicol Sci.

[r10] European Food Safety Authority (2009). Scientific opinion of the panel on contaminants in the food chain on a request from the European Commission on cadmium in food.. EFSA J.

[r11] Galicia-Garcia V, Rojas-Lopez M, Rojas R, Olaiz G, Rios C. (1997). Cadmium levels in maternal, cord and newborn blood in Mexico City.. Toxicol Lett.

[r12] Geary MP, Pringle PJ, Rodeck CH, Kingdom JC, Hindmarsh PC (2003). Sexual dimorphism in the growth hormone and insulin-like growth factor axis at birth.. J Clin Endocrinol Metab.

[r13] Gwatkin DR, Rustein S, Pande J, Wagstaff RP (2000). Socio-economic Differences in Health, Nutrition, and Population in Bangladesh. Washington, DC:World Bank.. http://siteresources.worldbank.org/INTPAH/Resources/Publications/Country-Reports/bangladesh.pdf.

[r14] Henson MC, Chedrese PJ (2004). Endocrine disruption by cadmium, a common environmental toxicant with paradoxical effects on reproduction.. Exp Biol Med (Maywood).

[r15] Hernandez M, Schuhmacher M, Fernandez JD, Domingo JL, Llobet JM (1996). Urinary cadmium levels during pregnancy and postpartum. A longitudinal study.. Biol Trace Elem Res.

[r16] Ikeda M, Watanabe T, Ohashi F, Shimbo S. (2010). Effects of variations in cadmium and lead levels in river sediments on local foods and body burden of local residents in non-polluted areas in Japan.. Biol Trace Elem Res.

[r17] Jameson S. (1993). Zinc status in pregnancy: the effect of zinc therapy on perinatal mortality, prematurity, and placental ablation.. Ann NY Acad Sci.

[r18] Järup L, Åkesson A. (2009). Current status of cadmium as an environmental health problem.. Toxicol Appl Pharmacol.

[r19] Johnson MD, Kenney N, Stoica A, Hilakivi-Clarke L, Singh B, Chepko G (2003). Cadmium mimics the *in vivo* effects of estrogen in the uterus and mammary gland.. Nat Med.

[r20] Khan NI, Bruce D, Naidu R, Owens G (2009). Implementation of food frequency questionnaire for the assessment of total dietary arsenic intake in Bangladesh: part B, preliminary findings.. Environ Geochem Health.

[r21] Kippler M, Ekström EC, Lönnerdal B, Goessler W, Åkesson A, El Arifeen S (2007). Influence of iron and zinc status on cadmium accumulation in Bangladeshi women.. Toxicol Appl Pharmacol.

[r22] Kippler M, Hoque AM, Raqib R, Öhrvik H, Ekström EC, Vahter M (2010a). Accumulation of cadmium in human placenta interacts with the transport of micronutrients to the fetus.. Toxicol Lett.

[r23] Kippler M, Lönnerdal B, Goessler W, Ekström EC, Arifeen SE, Vahter M (2009). Cadmium interacts with the transport of essential micronutrients in the mammary gland—a study in rural Bangladeshi women.. Toxicology.

[r24] Kippler M, Nermell B, Hamadani J, Tofail F, Moore S, Vahter M. (2010b). Burden of cadmium in early childhood: longitudinal assessment of urinary cadmium in rural Bangladesh.. Toxicol Lett.

[r25] Kuhnert PM, Kuhnert BR, Erhard P, Brashear WT, Groh-Wargo SL, Webster S (1987). The effect of smoking on placental and fetal zinc status.. Am J Obstet Gynecol.

[r26] Lin CM Doyle P Wang D Hwang YH Chen PC 2010 Does prenatal cadmium exposure affect fetal and child growth? Occup Environ Med; doi: [Online 23 December 2010] 10.1136/oem.2010.059758 21186202

[r27] Llanos MN, Ronco AM (2009). Fetal growth restriction is related to placental levels of cadmium, lead and arsenic but not with antioxidant activities.. Reprod Toxicol.

[r28] McElroy JA, Shafer MM, Hampton JM, Newcomb PA (2007). Predictors of urinary cadmium levels in adult females.. Sci Total Environ.

[r29] McTernan CL, Draper N, Nicholson H, Chalder SM, Driver P, Hewison M (2001). Reduced placental 11beta-hydroxysteroid dehydrogenase type 2 mRNA levels in human pregnancies complicated by intrauterine growth restriction: an analysis of possible mechanisms.. J Clin Endocrinol Metab.

[r30] Mueller BR, Bale TL (2008). Sex-specific programming of offspring emotionality after stress early in pregnancy.. J Neurosci.

[r31] Murphy VE, Gibson PG, Giles WB, Zakar T, Smith R, Bisits AM (2003). Maternal asthma is associated with reduced female fetal growth.. Am J Respir Crit Care Med.

[r32] Murphy VE, Smith R, Giles WB, Clifton VL (2006). Endocrine regulation of human fetal growth: the role of the mother, placenta, and fetus.. Endocr Rev.

[r33] Nermell B, Lindberg AL, Rahman M, Berglund M, Persson LA, El Arifeen S (2008). Urinary arsenic concentration adjustment factors and malnutrition.. Environ Res.

[r34] Nishijo M, Nakagawa H, Honda R, Tanebe K, Saito S, Teranishi H (2002). Effects of maternal exposure to cadmium on pregnancy outcome and breast milk.. Occup Environ Med.

[r35] Nishijo M, Satarug S, Honda R, Tsuritani I, Aoshima K. (2004a). The gender differences in health effects of environmental cadmium exposure and potential mechanisms.. Mol Cell Biochem.

[r36] Nishijo M, Tawara K, Honda R, Nakagawa H, Tanebe K, Saito S. (2004b). Relationship between newborn size and mother’s blood cadmium levels, Toyama, Japan.. Arch Environ Health.

[r37] Odland JO, Nieboer E, Romanova N, Thomassen Y, Lund E (1999). Blood lead and cadmium and birth weight among sub-arctic and arctic populations of Norway and Russia.. Acta Obstet Gynecol Scand.

[r38] Olsson IM, Bensryd I, Lundh T, Ottosson H, Skerfving S, Oskarsson A (2002). Cadmium in blood and urine—impact of sex, age, dietary intake, iron status, and former smoking—association of renal effects.. Environ Health Perspect.

[r39] Ooi SK Wolf D Hartung O Agarwal S Daley GQ Goff SP 2010 Dynamic instability of genomic methylation patterns in pluripotent stem cells. Epigenetics Chromatin 3 17; doi:10.1186/1756-8935-3-17 [Online 24 September 2010] 20868487 PMC2954997

[r40] Osman K, Åkesson A, Berglund M, Bremme K, Schutz A, Ask K (2000). Toxic and essential elements in placentas of Swedish women.. Clin Biochem.

[r41] Rahman A, Vahter M, Smith AH, Nermell B, Yunus M, El Arifeen S (2009). Arsenic exposure during pregnancy and size at birth: a prospective cohort study in Bangladesh.. Am J Epidemiol.

[r42] Rogers JM (2009). Tobacco and pregnancy.. Reprod Toxicol.

[r43] Ronco AM, Arguello G, Munoz L, Gras N, Llanos M (2005). Metals content in placentas from moderate cigarette consumers: correlation with newborn birth weight.. Biometals.

[r44] Ronco AM, Urrutia M, Montenegro M, Llanos MN (2009). Cadmium exposure during pregnancy reduces birth weight and increases maternal and foetal glucocorticoids.. Toxicol Lett.

[r45] Salpietro CD, Gangemi S, Minciullo PL, Briuglia S, Merlino MV, Stelitano A (2002). Cadmium concentration in maternal and cord blood and infant birth weight: a study on healthy non-smoking women.. J Perinat Med.

[r46] Seckl JR, Holmes MC (2007). Mechanisms of disease: glucocorticoids, their placental metabolism and fetal “programming” of adult pathophysiology.. Nat Clin Pract Endocrinol Metab.

[r47] Senn M, Baiwog F, Winmai J, Mueller I, Rogerson S, Senn N. (2009). Betel nut chewing during pregnancy, Madang province, Papua New Guinea.. Drug Alcohol Depend.

[r48] Shirai S Suzuki Y Yoshinaga J Mizumoto Y 2010 Maternal exposure to low-level heavy metals during pregnancy and birth size. J Environ Sci Health A Tox Hazard Subst Environ Eng 45:1468–1474. 10.1080/10934529.2010.500942 20694885

[r49] Straif K, Benbrahim-Tallaa L, Baan R, Grosse Y, Secretan B, El Ghissassi F (2009). A review of human carcinogens—part C: metals, arsenic, dusts, and fibres.. Lancet Oncol.

[r50] Thompson J, Bannigan J. (2008). Cadmium: toxic effects on the reproductive system and the embryo.. Reprod Toxicol.

[r51] Tofail F, Persson LA, El Arifeen S, Hamadani JD, Mehrin F, Ridout D (2008). Effects of prenatal food and micronutrient supplementation on infant development: a randomized trial from the Maternal and Infant Nutrition Interventions, Matlab (MINIMat) study.. Am J Clin Nutr.

[r52] Turgut S, Kaptanoglu B, Turgut G, Emmungil G, Genc O. (2005). Effects of cadmium and zinc on plasma levels of growth hormone, insulin-like growth factor I, and insulin-like growth factor-binding protein 3.. Biol Trace Elem Res.

[r53] Vahter M, Åkesson A, Liden C, Ceccatelli S, Berglund M. (2007). Gender differences in the disposition and toxicity of metals.. Environ Res.

[r54] Vahter ME, Li L, Nermell B, Rahman A, El Arifeen S, Rahman M (2006). Arsenic exposure in pregnancy: a population-based study in Matlab, Bangladesh.. J Health Popul Nutr.

[r55] Vatten LJ, Nilsen ST, Odegard RA, Romundstad PR, Austgulen R (2002). Insulin-like growth factor I and leptin in umbilical cord plasma and infant birth size at term.. Pediatrics.

[r56] Wier PJ, Miller RK, Maulik D, DiSant’Agnese PA (1990). Toxicity of cadmium in the perfused human placenta.. Toxicol Appl Pharmacol.

[r57] Yang K, Julan L, Rubio F, Sharma A, Guan H. (2006). Cadmium reduces 11 beta-hydroxysteroid dehydrogenase type 2 activity and expression in human placental trophoblast cells.. Am J Physiol Endocrinol Metab.

[r58] Zhang YL, Zhao YC, Wang JX, Zhu HD, Liu QF, Fan YG (2004). Effect of environmental exposure to cadmium on pregnancy outcome and fetal growth: a study on healthy pregnant women in China.. J Environ Sci Health Part A Tox Hazard Subst Environ Eng.

